# Redundancy in Growth Factor Receptor Signaling in Adult Astrocytoma Resistance to Small-Molecule Tyrosine Kinase Inhibitors

**DOI:** 10.3390/ijms27031196

**Published:** 2026-01-24

**Authors:** Roxana Radu, Anica Dricu, Ligia Gabriela Tataranu, Oana Alexandru

**Affiliations:** 1 Department of Neurosurgery, Faculty of Medicine, “Carol Davila” University of Medicine and Pharmacy, 020022 Bucharest, Romania; 2 Department of Biochemistry, Faculty of Medicine, “Carol Davila” University of Medicine and Pharmacy, 020022 Bucharest, Romania; 3 Department of Neurosurgery, Clinical Emergency Hospital “Bagdasar-Arseni”, 041915 Bucharest, Romania; 4 Department of Neurology, Faculty of Medicine, University of Medicine and Pharmacy, 200349 Craiova, Romania; oanale@hotmail.com

**Keywords:** adult astrocytoma, glioblastoma, tyrosine kinase inhibitors, drug resistance, growth factor receptors, EGFR, signaling pathways, targeted therapy

## Abstract

Adult astrocytomas, particularly IDH1/IDH2-wildtype infiltrating astrocytic gliomas, represent a significant challenge for medical professionals. Despite recent progress in understanding tumor biology and the use of molecular biomarkers, therapeutic options have not significantly improved patient outcomes. Although targeted therapies, such as small-molecule tyrosine kinase inhibitors (TKIs), have shown benefits in other solid tumors, they have largely failed to improve survival in adult astrocytoma patients. Characterized by remarkable heterogeneity, these tumors develop robust drug resistance mechanisms. The molecular processes driving this resistance are complex and not yet fully understood. In this review, we briefly present the growth factor receptors (GFRs) and their signaling pathways in adult astrocytomas and discuss the known mechanisms of resistance to small-molecule tyrosine kinase inhibitors.

## 1. Introduction

Brain tumors (BTs), which are the result of an uncontrolled proliferation of abnormal cells, still represent a challenge for patients and medical professionals. According to their nature, they can be classified as benign or malignant. However, the World Health Organization (WHO) established the most accurate classification. In accordance with this grading, BTs can rank from 1 to 4 [1]. The first two grades, 1 and 2, also known as low-grade BTs, are considered benign. On the other hand, grades 3 and 4 are malignant [1]. Among them are adult astrocytomas, the most common primary BT. The new 2021 WHO classification is a consequence of a better understanding of central nervous system (CNS) tumor biology. The molecular diagnosis of BTs is a result of intense research on cancer genomics, and although the therapeutic options for these patients are still limited, the innovations in targeted therapies have brought hope to these patients. However, diagnostic and treatment challenges remain because more than 100 primary brain tumors have been described [2]. Regarding CNS adult diffuse gliomas, this new classification brought an important change in infiltrating glioma definition. Therefore, glioblastoma (GBM) is an *IDH1*/*IDH2*-wildtype infiltrating astrocytic glioma in adults. In recent years, adult astrocytoma therapeutic methods have progressed due to advances in surgical methods, personalized radiotherapy, chemotherapy, and immunotherapy plans [3]. Despite all this progress, however, there has only been a small increase in median free-progression survival, especially for GBM patients, and the quality of life of these patients remains poor [4]. One explanation for this therapeutic failure is the presence of drug resistance. This can be explained by the heterogeneity of adult astrocytomas, especially GBM.

Growth factors (GFs) such as epidermal growth factor (EGF), platelet-derived growth factor (PDGF), vasculo-endothelial growth factor (VEGF), insulin-like growth factor (IGF), fibroblast growth factor (FGF), neurotrophins, and their receptors are involved in the development of both nonneoplastic and neoplastic cells of the CNS [5]. CNS cell proliferation, division, and differentiation are the result, among other processes, of GF binding to their corresponding receptors and the activation of intracellular signaling pathways. All these pathways determine cascade events, which finally modulate cells’ fundamental functions like proliferation, differentiation, growth, motility, metabolism, survival, and apoptosis [6]. Signaling pathways like phosphatidylinositol-3 (PI-3) kinase/protein kinase B(Akt)/mammalian target of rapamycin (mTOR) [7] or mitogen-activated protein kinase (MAPK)/extracellular-signal-regulated kinases (ERKs) [8], along with protein kinase C (PKC) [9] and Janus kinase (JAK)/signal transducer and activator of transcription (STAT) [10], are dominant pathways involved in GF signaling. An important observation is that these signaling pathways can influence, interact, and reinforce each other [11]. When the normal equilibrium between glioma cell proliferation, differentiation, survival, and apoptosis is disrupted in astrocytic cells, the consequence is glioma formation. A consequence of the genetic mutations in gliomas is the overproduction of GFs or of GFRs. This event determines which CNS cells will be in continuous proliferation. A sensible way to combat this phenomenon is to inhibit GFs or GFRs. However, there is more than one GF or GFR. Therefore, another solution is to inhibit at different key points the GF intracellular signaling pathway [6].

In recent years, a new class of specific drug that targets intracellular signaling has joined classical glioma treatment, represented by surgery, radiotherapy, and chemotherapy. These drugs are molecular targeted therapies and among them are small-molecule tyrosine kinase inhibitors (TKIs). They are among the most important drug targets discovered in the past century. There are two classes: those that target the ATP binding site and allosteric kinase inhibitors. Currently, there are 80 FDA-approved small-molecule tyrosine kinase inhibitors, and 180 drugs are being tested in clinical trials [12]. Some of these inhibitors are considered attractive targets for the treatment of adult astrocytomas. However, even if they have sometimes had promising results in preclinical studies, unfortunately in clinical trials the results are disappointing. Usually, this type of drug targets either one or more protein kinases. The explanation for their failure in adult astrocytoma patients may be the tumor’s resistance to treatment. Five mechanisms that explain this phenomenon are discussed by specialists: the presence of tumor mutations, the coactivation of other RTKs, the adaptation of glioma cells to inhibitors, the activation of compensatory intracellular signaling pathways, and the toxic effect of glioma stem cells on small-molecule protein kinase inhibitors [13].

The mechanisms involved in the resistance to TKIs are activation of alternative routes, coactivation, adaptation, and specific mutations [14]. In recent years, the linear model (pre-existent clones) and the divergent model (small-molecule tyrosine kinase inhibitors inducing changes in glioma cells) have been most prominently discussed by scientists [15]. Acquired alterations are also involved in tumor heterogeneity, which seems to explain the fact that resistant clones may induce resistance to treatment [16]. In GBM, an important degree of plasticity as a consequence of glioma stem cells was observed, which can also give rise to variable progeny, a phenomenon capable of explaining the change in glioma subtype [17,18]. This plasticity of glioma stem cells is influenced by cell surface regulators like EGF, which bind EGFR, which, in turn, plays an important role in the glioma stem cell phenotype; moreover, EGFR signaling is important for glioma stem cell maintenance [19]. These cells are also maintained by autocrine TGF-β signaling. The Notch signaling pathway is capable of regulating neural stem cells, the inhibition of which may promote sensitivity to therapy. The SHH pathway is also involved in neural stem biology through binding to the Patched receptor and the activation of GL1 transcription factors. Among the intracellular glioma stem cell regulators involved in plasticity are the PI3K/PTEN/Akt/mTOR pathway, NF-κB, and STAT3, which are involved in neural stem cell regulation, differentiation, and proliferation [20]. Therapeutic resistance can be a consequence either of tumor resection, which can enhance stem cell properties, or chemoradiation, which can also stimulate the development of therapy-resistant subclones. Tumor resection can enhance stem cell properties either through an incomplete resection (infiltrative tumors) or through protective niches (vascular niches). Incomplete resection promotes tumor heterogeneity and quiescence through resilient glioma stem cells. Another possibility is therapeutic evasion because glioma stem cells are characterized by resistance mechanisms like slow division, DNA repair, and metabolic adaptation [21]. Because single-agent therapy with TKIs has proved not to be efficient in adult astrocytomas, researchers are testing drug combinations. These are targeting alternative survival routes like EGFR/MET/VEGF pathways, using multi-kinase inhibitors (e.g., Foretinib or Dacomitinib) alone or in combination with anti-angiogenics (Onartuzumab with Bevacizumab), integrating checkpoint inhibitors (Nivolumab), combining kinome reprogramming targets with TKIs, or using natural compounds (phytotherapy) alongside drugs. These methods aim at overcoming bypass mechanisms and improving efficacy beyond single-agent TKIs [22].

The tumor microenvironment is a complex niche formed of endothelial cells, neurons, astrocytes, oligodendrocytes, resident immune cells, tumor-infiltrating circulating immune cells, noncellular components, exosomes, extracellular matrix (ECM) components, and secreted ECM remodeling enzymes [23]. It plays a crucial role in adult astrocytoma cell survival and response to therapies [24]. Due to GBM neovascularization, there are intratumoral variances in vascular and metabolic supply, including oxygen. This irregular vascular supply, with increased oxygen consumption by tumoral cells, generates tissue hypoxia, which results in an increased glucose uptake and glycolysis. This phenomenon is known as the “Warburg effect” and is independent of the oxygen uptake [25]. Another metabolic process which is able to supply energy to stimulate rapid tumor proliferation and survival is linked to HIF-1. Tissue hypoxia activates the STAT3 pathway, followed by HIF-1α synthesis, which stimulates the production of growth factors like VEGF which are involved in tumor angiogenesis [26]. In the end, this entire process supplies energy and nutrients for rapid genome replication [27]. This is another factor that can explain GBM resistance to treatment [28].

The blood–brain barrier (BBB)’s role in TKI penetration and therefore efficiency in adult astrocytomas has been intensely discussed. This neuroprotective barrier, formed by a monolayer of endothelial cells tightly interconnected with adherent junctions and tight junctions, restricts the passage of harmful substances into the brain [29], with 98% of the small molecules being unable to penetrate the BBB [30].

In adult astrocytomas, especially in GBM, the BBB is disrupted due to the infiltration of tumor cells and the overexpression of VEGF, which promotes angiogenesis and the leakage of the barrier. However, in the peritumoral zone, the BBB can be intact, forming the blood–brain tumor barrier (BBTB), which limits the entrance of drugs into the tumor [31]. An important observation is that TKIs must reach their therapeutic concentration at the tumor site. Deficient penetration of the BBB seriously hampers their efficacy in adult astrocytomas [32]. Therefore, it is extremely important to select TKIs with good BBB penetrability or to develop novel methods of drug delivery into the tumor for GBM patients.

Here, we intend to present the involvement of GFR signaling in the progression of adult astrocytomas as well as the mechanisms of resistance to targeted therapies.

## 2. The 2021 Adult Diffuse Glioma Classification

The traditional classification of adult diffuse gliomas has been mainly based on tumor histology. Once molecular mechanisms and genetic research developed, a new classification in 2016, the fourth edition of the WHO classification of CNS tumors, took into consideration the Isocitrate Dehydrogenase 1 and 2 (IDH1 and IDH2) mutations. Since then, IDH status determination has represented the basis of diffuse adult glioma diagnosis [33]. The new information, obtained from advances leading to a better understanding of the molecular pathogenesis of adult diffuse gliomas, is now incorporated in the fifth edition of the WHO classification of CNS tumors [2].

Since 2021, adult diffuse gliomas have been classified into three tumor types: astrocytoma, IDH-mutant; oligodendroglioma, IDH-mutant and 1p/19q-codeleted; and glioblastoma, IDH-wildtype. In accordance with their histological and molecular characteristics, IDH-mutant astrocytomas are WHO grade 2, 3, or 4. Oligodendrogliomas IDH-mutant and 1p/19q-codeleted are WHO grade 2 or 3. Glioblastomas are *IDH1*/*IDH2*-wildtype infiltrating astrocytic gliomas in adults, characterized by microvascular proliferation or necrosis, and further by TERT-mutation, and/or EGFR-amplification, and/or 7q/10 chromosome loss, and are WHO grade 4 [2].

## 3. GFRs in Adult Astrocytomas

GFs are molecules capable of influencing the process of mitosis (either stimulation or inhibition) and also interfere with cellular differentiation. These molecules target specific receptors which are expressed on the surface of cells. GFRs are membrane-bound enzyme-like receptors, and most of them are receptor tyrosine kinases (RTKs). The structure of these receptors consists of an extracellular domain, also known as ligand (GF)-binding domain, a transmembrane domain, a cytoplasmic domain that acts as an enzyme, and some tyrosine residues that act like docking sites for the phosphorylated cytoplasmic signaling effectors. GF binding to their cognate receptors leads to the phosphorylation of tyrosine residues and the activation of several signal transduction pathways which are involved in proliferation, survival, invasiveness, and angiogenesis [34].

GFs play an important role in the growth of normal CNS cells but also of adult astrocytomas. Among them, EGF, IGF, PDGF, FGF, VEGF, and their receptors are involved in normal CNS development [5]. In this situation, the activity of RTKs is controlled. Therefore, RTK signaling controls processes like the growth, survival, differentiation, and migration of CNS cells.

When various mutations are present, there is an overexpression or amplification of RTKs, mutations of the ligand-binding domain or kinase domain, fusion of kinase domains with other proteins, or secretion of a chimeric product with intense kinase activity. All that leads to the uncontrolled activation of signaling pathways [35]. Throughout the activation of these signaling pathways, TKs may become important promoters of tumor growth. Because this situation maintains the cells in a state of abnormal, persistent proliferation, it will lead them to uncontrolled tumorigenesis.

Glial cells are characterized by a dichotomy between proliferation and invasion, which in the end determines the nature of tumor progression [36]. The progression of tumor cells is also influenced by microenvironmental changes [37].

Although until now 58 RTKs have been identified in humans, only some of them are frequently altered in BTs. Among them are EGFR, IR, PDGFR, VEGFR, FGFR, MET, and Tie receptors.

### 3.1. EGFR in Adult Astrocytomas

EGFR is a receptor family that is composed of four transmembrane receptors: EGFR(ErB1), ErbB2/HER2, ErbB3/HER3, and ErbB4. It plays an important role in processes like the proliferation, survival, and differentiation of CNS cells [38].

The receptor is activated by ligands. After activation, it undergoes dimerization, followed by the trans-autophosphorylation of the intracellular domain and the activation of downstream signaling pathways. The overexpression or mutation of EGFR activates intracellular signaling pathways like PI3K/Akt, Ras/Raf/Mek/ERK, or Jak/STAT [39,40].

In glioma, EGFR is frequently amplified and associated with aggressive behavior. For instance, in GBM, the receptor is overexpressed in about 60% of patients [41,42,43]. Coexpression of EGFR and EGFR*vIII* is also frequent in astrocytic gliomas, particularly GBM. EGFR*vIII* is a structural alteration characterized by the deletion of exons 2 to 7 from the extracellular domain. It has some particular characteristics like its activation, which is independent from its ligand, or the fact that it is constitutively phosphorylated [44]. Coexpression with EGFR leads to its phosphorylation, which furthermore activates the STAT/5 pathway through fibroblast growth factor-inducible 14 (Fn14) and stimulates malignant progression [45]. In IDH-mutant astrocytomas, EGFR amplification is not taken into consideration when establishing a diagnosis [2]. Although amplification is rather rare in this type of gliomas, it is associated with a more aggressive behavior and worse survival [46].

Regarding the other EGF receptors, researchers have demonstrated the elevated levels of Erb2 and Erb3 in gliomas, especially GBM [47,48], and their association with a poor prognosis.

EGFR small-molecule inhibitors have shown promising results in preclinical studies of glioma cell lines.

In recent years, several generations of small-molecule inhibitors of EGFR have developed. First-generation drugs like Erlotinib or Gefitinib seemed efficient when tested in GB cell lines [49], but failed when tested in clinical trials, either alone or in combination with other therapies [50]. While the results of second-generation small-molecule inhibitor Afatinib were limited when used in monotherapy, its association with Temozolomide reduced the proliferation of GBM cells and had positive effects in mouse models of GBM [51]. A problem in the first two generations of EGFR inhibitors was their poor penetration of the blood–brain barrier (BBB). Drugs like Erlotinib, Gefitinib, Cediranib, and Vandetanib have a limited capacity to enter the BBB either due to P-glycoprotein or breast cancer resistance protein (ABCG2)-mediated active efflux (Table 1).

Regarding Nintedanib, the use of polymeric polyactic acid–glycolic acid-based nanoparticles for BBB delivery may improve the efficiency of the drug in GBM patients [56]. In clinical trials on adult astrocytoma patients, EGFR inhibitors like Gefitinib, Erlotinib, Cediranib, Vandetanib, Nintedanib, and Afatinib failed to improve overall survival [50,75,98,99,100].

This problem was solved with the third-generation EGFR small-molecule inhibitor Osimertinib, as preclinical tests that studied the drug alone and in combination were rather positive [101]. However, the effect of Osimertinib on GBM patients remains limited due to its inability to inhibit the extracellular domain of mutant EGFR [64].

Alongside tumor heterogeneity, poor tolerability, and difficulty in passing the BBB, a good explanation of tumor resistance to EGFR inhibitors is the compensatory changes in EGFR intracellular signaling pathways.

**Figure 1:**
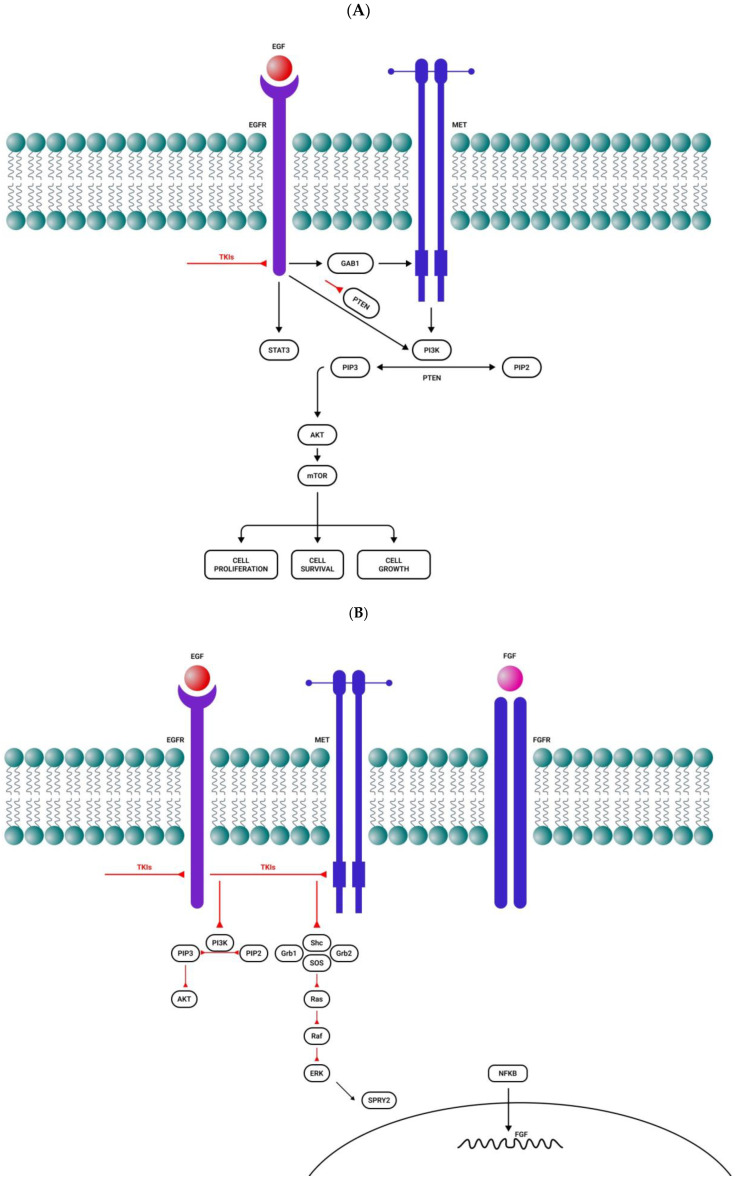

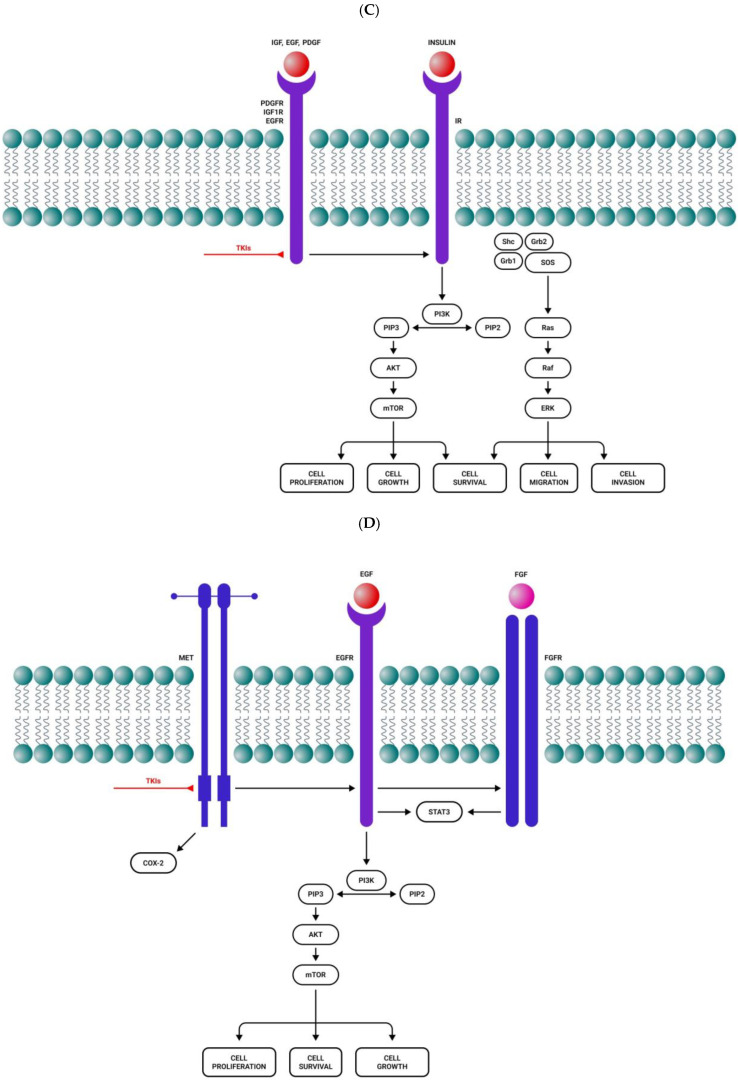
Activation of alternative roots after tyrosine kinase inhibitor administration. (**A**) After a short period of response to EGFR tyrosine kinase inhibitors, there is resistance to treatment by MET overactivation. (**B**) EGFR and MET inhibition activate the NF-kB pathway, followed by FGFR autocrine activation and cell survival. (**C**) Resistance to EGFR, PDGFR, and IGFR tyrosine kinase inhibitors by the compensatory secretion of IGF/insulin. (**D**) MET inhibition induces STAT3, EGFR, FGFR, and mTOR activation. Sharp arrows (→) represent stimulation while blunt arrows (┴) indicate inhibition.

After acquiring EGFR inhibitor resistance, GBM is capable of activating compensatory signaling pathways such as PI3K/Akt, Ras/Raf/Mek/ERK, or c-MET [102] (Figure 1A). IGFR1 overexpression also plays an important role in EGFR resistance through the PI3K/Akt signaling pathway [103] (Figure 1C). NF-Kb pathway activation may also be a consequence of both EGFR and c-MET inhibition, and it is followed by the autocrine activation of FGFR, which stimulates the survival of glial cells [104] (Figure 1B).

Specific mutations either of the drug target or other genes have also been discussed. Astrocytomas, especially GBM, are characterized by point mutations in the extracellular domain. Unfortunately, EGFR inhibitors such as Erlotinib or Gefitinib are designed to target the intracellular tyrosine kinase domain, while in adult astrocytomas, point mutations are mainly in the extracellular domain. This observation can explain the failure of treatment [105]. Lapatinib, an EGFR inhibitor which targets the extracellular domain, proved to be slightly better in a GBM clinical trial [106]. Moreover, these drugs are designed to target the active conformation of EGFR whereas in GBM the receptor has an inactive conformation [107].

The coactivation of other RTKs in adult astrocytomas, another possible cause of intrinsic tumor resistance to small-molecule tyrosine kinase inhibitors, is also explained at least in part by tumor heterogeneity. In fact, adult astrocytomas, especially GBM, were found to have tumor cells with only one RTK amplified while others are characterized by the overexpression of multiple RTKs [108]. For instance, some adult astrocytomas are characterized by the coactivation of EGFR, PDGFRα, and c-Met. Resistance to EGFR inhibitors is present in GBM characterized by EGFR/EGFRvIII activation and PTEN loss. c-MET, a promoter of tumor growth, is also involved in glioma resistance to EGFR inhibitors through the EGFRvIII–MET heterodimer, as well as through the activation of EGFR through an autocrine loop [102]. The overexpression of EGFRvIII suppresses the expression of PDGFRβ, but when the patient receives Erlotinib, an EGFR small-molecule kinase inhibitor, PDGFRβ, is reactivated [109].

Besides adult astrocytomas that do not express some RTKs and therefore do not respond to those RTK inhibitors, there are some adult astrocytomas which are characterized by an initial response to small-molecule kinase inhibitors, followed by an acquired resistance to treatment. The loss of extra-chromosomally encoded EGFR, involved in resistance to treatment, can be determined after Erlotinib treatment. After cessation of Erlotinib administration, the mutation reappears, affecting the treatment response [110].

Currently, fourth-generation EGFR small-molecule inhibitors are being tested in clinical trials of patients with GBM. BDTX-1535 is an oral, bioavailable, brain-penetrating, mutant-selective EGFR inhibitor that selectively targets and inhibits the activity of common EGFR mutations, as well as intrinsic and acquired resistance mutations. The inhibitor is currently being tested in clinical trials for GBM patients [58,65]. In a preclinical study, it proved to be very potent and selective against a broad range of extracellular domain mutations and amplified EGFR and wildtype EGFR sparing [111].

Another selective EGFR inhibitor with high BBB penetration is ERAS-801, which is currently being tested in clinical trials for GBM patients and has already demonstrated good safety and tolerability [59].

### 3.2. IGFR1 in Adult Astrocytomas

The two surface insulin receptors, IR1 and IR2, are part of the IGF axis along with the two ligands, IGF1 and IGF2, the binding proteins IGFBP1-6, and their degrading enzymes. IGFR1, the main mediator of IGF1 and IGF2, is also linked to downstream signaling pathways like PI3K/Akt and Ras/Raf/Mek/ERK. IGF1 and IGF2 along with IGFR1 play important roles in the development and growth of glial cells [5]. The receptor is expressed in high amounts in the human brain and is capable of regulating processes like cell proliferation and differentiation. It is overexpressed in adult astrocytomas [112,113]. IGF1R can also be used as a biomarker in GBM patients. This is because the receptor’s overexpression is associated with tumor resistance to temozolomide treatment and also with a reduced overall survival of GBM patients [113]. The overexpression of IGF1R stimulates GBM cell proliferation through the PI3K/Akt/mTOR signaling pathway, which is also involved in drug resistance [114]. In 2020, Simpson et al. demonstrated that the re-enhanced expression of IGFR1 is associated with a poor prognosis in adult astrocytoma patients [115]. In 2022, Martin et al. reported that the nuclear localization of IGFR1 increases GBM cell motility and metabolism. The same authors also observed a translocation of IGFR1 to the nucleus in vivo, which induced an increase in GBM proliferation rate [116].

Targeting of IGFR1 has already proved effective on adult astrocytoma cells [117,118]. Linsitinib also decreased the in vitro viability of GBM cells [119] and its antiproliferative effect was higher than that of BMS-754807 [120]. In general, the results of IGFR1 inhibitor treatment were rather modest. One explanation of adult astrocytomas’ resistance to IGFR1 small-molecule inhibitors could be alternative RTK activation. For instance, it has been demonstrated that the activation of the EGFR pathway contributes to IGFR1 drug resistance in various tumors [121]. Moreover, the activation of IGFR1 signaling pathways like PI3K/Akt, Ras, and STAT3 may confer resistance to IGFR1 inhibitors. Another explanation is the compensatory secretion of IGF/insulin determined by IGFR1 inhibition. In fact, there is an intrinsic and an acquired resistance to anti-IGFR1 therapies conferred by IR [122]. It has been observed that the inhibition of both IGFR1 and IR may increase anti-tumor efficacy, and therefore dual inhibitors like BMS-754807 or Linsitinib should be more effective [119]. Moreover, anti-IGFR1 inhibitors are capable of stimulating the tumor microenvironment to secrete IGF. This IGF can activate alternate signaling pathways, contributing to resistance to anti-IGFR1 drugs [123].

In adult astrocytoma cells, the IGF pathway is involved in acquired resistance to RTKIs through various mechanisms, one of which is the activation of alternative RTK compensatory signaling pathways. It is already known that IGFR1 cross-talks with several receptors like EGFR, ErB2, ErB3, and MET. Among their signaling transduction pathways, these receptors have common nodes. Therefore, when one receptor is inhibited, another may compensate its activity [124]. For instance, when treatment with small-molecule kinases inhibits the EGFR pathway, there is an alternative activation of the IGF pathway, which, in turn, activates the PI3K/Akt and MAPK/ERK signaling pathways downstream [125] (Figure 1C). Considering this observation, the combined therapy with IGF/IGFR1 and EGFR inhibitors seems a promising option that could combat adult astrocytoma resistance to treatment. In preclinical studies, the combination of OSI-906 and Gefitinib was demonstrated to be a superior option to single therapy [124]. Similar results were obtained in a preclinical study that used AG1024 and AG1478 on human glioma cells [126].

In other glial tumors, PDGFR resistance to treatment is associated with the overexpression of IGFR1 [127] (Figure 1C).

### 3.3. PDGFR in Adult Astrocytomas

PDGFRs are normally involved in the development of embryos, cellular growth, differentiation, and blood vessel formation (PDGFRβ). The receptors are expressed in the CNS, especially in oligodendrocytes (PDGFRα) [5]. Other receptors of the same ligand are stem cell factor (SCF) receptor/CD117 (c-KIT) and Fms-like tyrosine kinase 3 (Flt3). c-KIT is normally expressed in the CNS and promotes angiogenesis, while Flt3 is normally expressed in progenitor cells [128,129]. Ligand binding is followed by the dimerization of the receptor and the transphosphorylation of the intracellular domains, which ends with receptor activation. This process is followed by the downstream activation of intracellular signaling pathways like Ras/Raf/Mek/ERK and PI3K/Akt.

Overexpression of PDGFRs in adult astrocytomas is frequently encountered. PDGFRα and PDGFRβ expression is higher in GBM when compared with IDH-mutant adult diffuse astrocytomas [130]. Moreover, in these tumors, both the autocrine and paracrine loops that activate the PDGF system seem to be present [131].

c-KIT is also overexpressed in IDH-mutant astrocytomas as well as in GBM [128]. It was also observed that the receptor’s overexpression is not associated with c-KIT gene amplification [132].

As a result, PDGF and PDGFRs are considered potential therapeutic targets for adult astrocytoma patients. Unfortunately, their effect either alone or in combination with other agents on high-grade glioma (HGG) cells was rather modest [133,134]. In clinical trials, Gleevec, either alone [68] or in combination with Nilotinib (a PDGFR and c-Kit inhibitor), had limited effects when administered to GBM patients [69]. Tandutinib (a PDGFR, Flt-3, and c-Kit inhibitor) in association with Bevacizumab did not improve the situation in patients with recurrent GBM and had important adverse effects in a phase II clinical trial [70]. Because the BBB penetrability of these drugs is significant (Table 1), one explanation is that PDGFR and c-KIT frequently target mutations in the intracellular domain, while adult astrocytomas are characterized by mutations in the extracellular domain. Therefore, PDGFR inhibitors are also inefficient for adult astrocytomas with these mutations [135]. Another explanation for adult diffuse gliomas’ resistance to PDGFR inhibitors is the coactivation of alternative RTKs like ERBB3, IGFR1, and TGFBR2 [136].

### 3.4. VEGFR in Adult Astrocytomas

VEGFR1 (Flt1), VEGFR2 (Flk1 or Kdr), and VEGFR3 (Flt4) are normally expressed in vascular endothelial cells, monocytes, macrophages, and lymphatic cells [137]. The VEGF axis promotes CNS angiogenesis, increases BBB permeability, and is involved in the inflammatory processes of the brain [138]. While VEGFR1 is involved in vascular formation, VEGFR2 controls the differentiation of endothelial cells and blood vessel formation [139]; ligand binding is followed by receptor autophosphorylation, which activates intracellular signaling pathways downstream.

Adult astrocytomas are highly vascularized and therefore express high levels of VEGF, especially VEGFA and cognate receptors. For instance, GBM is characterized by increased angiogenesis with aberrant vascularization, processes that are a consequence of VEGFR2 overexpression in GBM cells [140]. It is known that VEGFR2 is a key regulator of proliferation, migration, survival, and vascular permeability; at high levels, it confers resistance to therapy and stimulates GBM proliferation [141].

The most studied VEGF inhibitor, approved by the FDA, is Bevacizumab, also known as Avastin, a monoclonal antibody with ineffective results either alone or in combination with other chemotherapeutic agents [142]. For years, various scientific groups have investigated VEGFR inhibitors in adult diffuse gliomas both in vivo and in vitro and also in clinical trials. Drugs like Axitinib, Cediranib, Pazopanib, Vandetanib, Sorafenib, and Sunitinib were tested in several types of cancer, including astrocytic tumors, both in vitro and in vivo [143,144,145].

The effect of these inhibitors was also tested in clinical trials on GBM patients, with a very modest improvement in the results when compared with conventional chemotherapy [77,146,147]. One explanation of adult astrocytomas’ resistance to anti-VEGFR therapy is the capacity of GBM cells to trigger alternative pathways. Another is their limited capacity to cross the BBB (Table 1). VEGFR inhibitors generate hypoxia which can also activate some alternative pathways to form new blood vessels through HIF-1α [22,148].

These tumors are capable of overexpressing alternative receptors and of activating alternative methods to promote tumor angiogenesis.

### 3.5. FGFR in Adult Astrocytomas

FGFR family members from 1 to 4 are involved in many cellular processes including proliferation, differentiation, migration, and survival [149]. FGFRs also play an important role in neurons (FGFR1) and glial cells (FGFR3). After birth, they influence a series of physiological events through the activation of fibroblast growth factor receptor substrate 2 (FRS2) or some downstream signaling pathways such as PI3K/Akt, MAPK/ERK1/2, STAT3, and phospholipase Cγ (PLCγ) [150]. These receptors are usually activated either by their ligands or by cell-adhesion molecules (CAMs). They undergo the process of dimerization, followed by the phosphorylation of tyrosine kinase domains. After kinase activation, downstream signaling pathways are activated, controlling cell growth and differentiation [151].

In adult astrocytomas, these receptors are overexpressed. For instance, FGFR1 and FGFR3 are overexpressed in WHO grade 2 IDH-mutant astrocytomas. In GBM, the overexpression of FGFR1 and FGFR2 was observed, which was also correlated with tumor progression [152]. However, FGFR3 and FGFR4 can also be expressed and correlate with glioma tumor invasiveness, a process stimulated by both autocrine and paracrine factors [153].

All these observations led to the idea that the inhibition of these receptors may improve the therapeutic response of adult diffuse glioma patients. Among the FGFR inhibitors studied in vitro, in vivo, and clinical trials are Fisogatinib (BLU-554), Ftibatinib, AZD4547, and Infigratinib. Only AZD4547 and Infigratinib have reached phase I and II clinical trials in GBM patients, and they exhibit a partial response [79], possibly due to their limited capacity to penetrate the BBB (Table 1).

### 3.6. Tunica Interna Endothelial Cell Kinase (Tie) Receptors in Adult Astrocytoma

Tie-1 and Tie-2 are the receptors of the Tie family, mainly expressed on the surface of endothelial cells. They control processes such as atherosclerosis, tumor angiogenesis, and vascular leakage in sepsis [80]. Tie-2 plays an important role in blood vessel development but also in the growth of tumor cells, promoting angiogenesis but also metastasis. Tie-2 signaling pathways are essential for proper brain angiogenesis and blood–brain barrier genesis during development [154].

Regarding adult diffuse glioma, Tie-2 is expressed in GBM cells and stimulates tumor progression. The receptor is capable of regulating the crosstalk between glioma cells and the tumor microenvironment [155]. Tie-2 activity in glioma has an agonist: Ang-2, the overexpression of which promotes glioma invasiveness through matrix metalloprotease 2 (MMP2) activation [156]. Therefore, the Ang-2/Tie-2 signaling pathway is used as a target to inhibit GBM growth and invasion. Rebastinib, a selective Tie-2 inhibitor, Bay-826, and Altiratinib inhibited tumor growth and limited invasiveness but with modest results [82], although they have a good capacity to cross the BBB (Table 1).

### 3.7. MET in Adult Astrocytomas

MNNG-HOS transforming gene/hepatocyte growth factor/HGF/c-MET is also known as an MET receptor or hepatocyte growth factor (HGF) receptor. Its ligand is HGF. The receptor is expressed in neuroblast precursors and myoblasts but also in endothelial and epithelial cells. The activation of MET is an important event in angiogenesis, proliferation, cancer invasion, and also wound healing [157]. During embryonic development, MET is expressed in the cortex as well as in the cerebellum. The ligand receptor determines the dimerization of MET, the transphosphorylation of tyrosine kinase residues, and, ultimately, the downstream activation of signaling pathways such as Ras/Raf/Mek/ERK and PI3K/Akt. PTEN and c-MET coregulate many genes which are involved in the regulation of cell growth [158].

**Figure 2:**
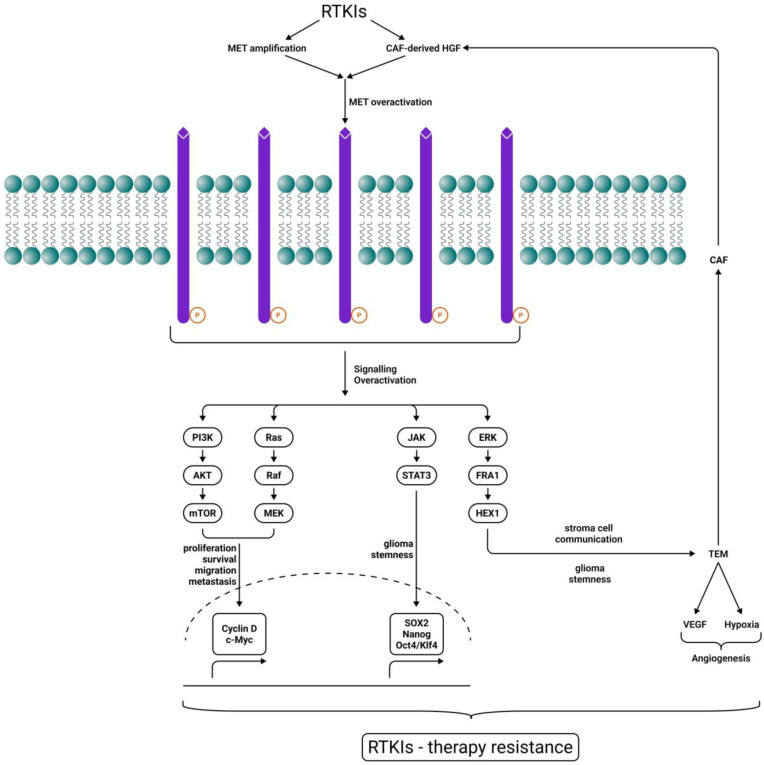
MET-driven RTKI therapy resistance. RTKIs induce CAF-derived hepatocyte growth factor (HGF) and MET overexpression, maintaining downstream signaling for tumor growth. Several MET intracellular signaling pathways, including PI3K/Akt/mTOR, RAS/RAF/MEK, JAK/STATs, and ERK/FRA1/HEY1, collaborate to induce cell proliferation and survival, migration, invasion, metastasis, angiogenesis, stromal cell communication, and cancer cell stemness, finally resulting in RTKI therapeutic resistance. Sharp arrows (→) indicate stimulation.

In GBM cells, this receptor is overexpressed and stimulates tumor growth, angiogenesis, but also invasion. The RTKI-induced MET/HGF pathway, MET amplification, and Cancer-Associated Fibroblast (CAF)-derived HGF expression are recognized as some of the most frequent and established adaptive mechanisms to bypass drug-induced signaling. They block several types of tumors, including gliomas. Several MET intracellular signaling pathways, including PI3K/Akt/mTOR, RAS/RAF/MEK, JAK/STATs, and ERK/FRA1/HEY1, collaborate to induce cell proliferation and survival, migration, invasion, metastasis, angiogenesis, stromal cell communication, and cancer cell stemness, finally resulting in RTKI therapeutic resistance [159] (Figure 2). Cabozantinib, a pan-TKI against MET, VEGFR2, RET, KIT, and FLT3, with a good capacity to cross the BBB (Table 1), was evaluated in phase I and II clinical trials with temozolomide and radiation therapy, but also in GBM patients who received prior antiangiogenic therapy. Although the drug had some clinical activity in patients with recurrent or refractory GBM, the results were not entirely satisfactory [85]. The use of MET inhibitor Crizotinib in GBM induces STAT3, the EGFR pathway, the FGFR pathway, and mTOR activation [160] (Figure 1D), although its capacity to penetrate the BBB is rather limited (Table 1).

## 4. Signal Transduction Pathways in Adult Astrocytomas

### 4.1. Ras/MAPK Pathway in Adult Astrocytomas

The MAPK pathway plays various physiological roles including cell differentiation, survival, gene expression, and regulation of blood glucose level. The proteins involved in this pathway are Ras, Raf, MEK, and ERK [161]. Its activation by RTKs starts with growth-factor-receptor bound-2 (GRB2), which forms a complex with Son of Sevenless (SOS), a guanine-nucleotide exchange factor (GEF). SOS1 plays an important role in Ras activation. Ras is a protein regulator which is capable of further activating Raf. Next, Raf promotes MEK which, in turn, activates ERK1/2. In the end, processes such as proliferation or apoptosis are modulated [161]. Dysregulation of the proteins involved in this pathway can lead to tumor progression. The overexpression of RTKs such as EGFR or FGFR that activate the Ras/MAPK pathway may cause uncontrolled cell growth. The most promising target for tumor therapy is MEK.

In adult astrocytomas, this pathway is often dysregulated [162]. For instance, in GBM, the Ras protein is activated. Ras activation in the CNS is a consequence of Ras mutation and determines the proliferation of tumor cells; it was described in adult astrocytoma cells, where it stimulated tumor progression from IDH-mutant to IDH-wildtype [163]. Ras alterations have also been observed in all types of adult astrocytomas. However, the mutation has a low incidence; in GBM, it is encountered in about 1% of cases [164]. Ras protein is also involved in the proliferation and angiogenesis of astrocytomas [165].

Phosphatase and TENsin homolog deleted on chromosome 10 (PTEN) is a tumor suppressor factor involved in cancer cell progression [166]. After binding and dephosphorylating the p52 isoform of Shc, phosphatase and TENsin homolog deleted on chromosome 10 (PTEN) determines the inhibition of Grb2 recruitment and the activation of the MAPK pathway. PTEN loss blocks EGF- and PDGF-induced MAPK activation in adult astrocytomas [167].

PTEN also can inhibit integrin- and growth factor-stimulated FAK (a key protein involved in the survival and migration of glioma cells) phosphorylation, also inhibiting tumor cell migration [168].

Shc is also capable of interacting with Gab1, a substrate of c-Met. Therefore, PTEN binding to Shc also inhibits c-Met-induced MAPK pathway activation [158].

RAS/MAPK signaling is negatively regulated by neurofibromin 1(NF1), which is a tumor suppressor. The loss of NF-1 is associated with increased levels of Ras-GTP. NF-1 negatively regulates RAS, converting the active RAS-guanozine triphosphate (RAS-GTP) into inactive RAS-guanozine diphosphate (RAS-GDP), which inhibits downstream RAS signaling [169]. Regarding GBM, the loss of NF-1 is found in about 15% of tumors and is associated with the mesenchymal type. This GBM type has a poor prognosis and is characterized by frequent recurrence [44].

It is also known that the mesenchymal type of GBM is a consequence of the transition from the proneural subtype, a phenomenon considered to be EMT-like. Because NF-1 is associated with the mesenchymal subtype of GBM, it can be considered a late event. In 2021, Marques et al. demonstrated that NF-1 is capable of regulating mesenchymal GBM plasticity and aggressiveness [170].

The MEK inhibitor Selumetinib was FDA-approved for the treatment of pediatric patients diagnosed with neurofibromatosis type 1 [171]. In preclinical studies and phase II clinical trials, the use of MEK inhibitors had only partial efficacy for GBM patients with NF-1 loss [172]. Selumetinib was also studied in pediatric patients with BRAF-aberrant or neurofibromatosis type 1-associated recurrent, refractory, or progressive low-grade glioma in a multicenter phase II trial [173]. However, the combined inhibition of BRAF and MEK had some efficacy in BRAF-mutant gliomas [125]. It is also known that NF-1 loss is associated with RAF inhibitor resistance in tumors [174], including GBM [175].

Since 2023, Trametinib has also been an FDA-approved MEK inhibitor for pediatric low-grade gliomas in association with Dabrafenib [8]. The drug has a limited capacity to cross the BBB, and its results are rather encouraging only when it is used in combination with Dabrafenib (Table 1). Various other inhibitors of the pathway are being tested in clinical trials, but the results are rather limited.

### 4.2. PI3K/Akt Pathway in Adult Astrocytomas

The PI3K/Akt/mTOR intracellular signaling pathway plays a very important role in the regulation of cellular processes like proliferation, growth, metabolism, apoptosis, and survival [176]. Ligand binding to RTK is followed by the autophosphorylation of the receptor. This activates PI3K, which adheres and phosphorylates a constituent of cell membrane PIP2 to PIP3. PIP3 binds to the PH domain, a process which is followed by Akt phosphorylation and activation by PDK1 [177]. TSG phosphatase TENsin homology (PTEN) has a central catalytic phosphatase core domain that negatively regulates PI3K through PIP3 and PIP2 dephosphorylation. PTEN is also capable of dephosphorylating other proteins like focal adhesion kinase (FAK), insulin receptor substrate 1, c-SRC, and even itself [178]. In turn, activated Akt can promote the phosphorylation of downstream effectors like mTOR and FOXO, but also inhibit the same process for GSK3, TSC2, or MDM2. Through this downstream regulation, Akt is involved in cell growth, survival, proliferation, and angiogenesis and is capable of inhibiting apoptosis [179].

In adult astrocytomas, it has been demonstrated that the PI3K/Akt pathway is activated in about 90% of GBM [180]. In IDH-wildtype infiltrating astrocytic gliomas, the PI3K/Akt pathway plays an important role in gliomagenesis, disease progression, and resistance to treatment. Moreover, the crosstalk between PI3K/Akt and other signaling pathways can over-activate mTOR in GBM. FilGAP, a small GTP-ase, hyperactivates Akt or translocation-associated membrane protein 2 (TRAM2) activates PI3K/Akt signaling, promoting tumor development [181,182]. Right open reading frame ½ (RIO1/2) stimulates the Akt/mTOR complex, consequently stimulating glioma migration and invasion [183].

PI3K/Akt/mTOR disruption is a hallmark of GBM. For patients with IDH-mutant low-grade gliomas, an increased activity of this pathway can be predictive of disease progression [184]. The PI3K/Akt/mTOR pathway is capable of influencing tumorigenesis, tumor growth, progression, tumor grade, resistance to treatment, and poor prognosis, and can be considered an important therapeutic target for adult astrocytoma patients.

The PI3K/Akt/mTOR pathway has the capacity of exhibiting intricate crosstalk with other signaling pathways in adult astrocytomas. The first one is the RAS–RAF–MAPK/ERK signaling pathway. For instance, mTOR is an intermediate between PI3K/Akt, MAPK, and PLCγ but also between PI3K/Akt and NF-κB. The inhibition of mTOR favors RTK internalization (especially EGFR), followed by downstream dysregulation of the MAPK/ERK signaling pathway. MAPK is also able to stimulate Akt/mTOR, thus stimulating GBM growth and migration [185].

In GBM, the Wnt/β-Catenin pathway can also be dysregulated. The crosstalk with PI3K/Akt signaling pathway is performed through Akt. PI3K suppresses Wnt/β-Catenin activity, stimulating tumor proliferation, but both pathways stimulate tumor survival [186].

Another mutual positive regulation has been reported between PI3/Akt/mTOR and the Sonic hedgehog pathway, stimulating GBM progression. Moreover, the interference between PI3K/Akt and Sonic hedgehog is correlated with tumor grade and survival [187].

Besides Wortmannin, a PI3K inhibitor which failed in clinical studies due to toxicity, other PI3K inhibitors which have been tested or are undergoing clinical trials either alone or in combination with other therapies are BKM120, PX-866, and Paxalisib [91,92,93,188], but the results are rather limited, although their capacity to cross the BBB is good (Table 1). Similar results were obtained when testing in clinical trials inhibitors of Akt, like Enzastaurin (Clinical Study NCT03776071) and PI3K/mTOR-like Bimiralisib (Clinical Study NCT02850744). mTOR inhibitors are the most studied. Therapeutic agents such as Temsirolimus (Clinical Study NCT03158389), Sirolimus (Clinical Study NCT00672243), and Everolimus (Clinical Study NCT01062399) have been tested either alone or in combination with other therapies in several clinical trials on GBM patients. The results are rather modest.

PTEN has the capacity of regulating PI3K/Akt/mTOR signaling, and PTEN alterations were observed in up to 40% of adult astrocytomas, most of them primary GBM [189]. Along with PTEN, other activating mutations were seen, namely, PI3K, and rarely Akt and mTOR [190]. RTKs activate PI3K, which through catalyzed phosphorylation generates active PIP3 and PIP2 and activates Akt downstream, which, in turn, activates mTOR. As a tumor suppressor, PTEN dephosphorylates PIP3 and PIP2 and, in this manner, prevents Akt activation [191].

Its partial loss or decrease in expression can lead to tumor progression through PI3K overactivation and PIP3 generation which stimulates Akt and mTOR [192]. Its signaling is very important in adult astrocytoma progression and also response to treatment. In fact, the inhibition of PTEN induces Akt signaling which, in turn, determines GBM proliferation, inhibition of apoptosis, and therapy resistance, with the latter being either to radiotherapy [193] or to chemotherapy, like temozolomide [194].

In gliomas, PTEN status was found to be a predictor of tumor response to RTK-targeted therapies. For instance, regarding EGFR small-molecule kinase inhibitors in GBM, tumors characterized by the coexpression of PTEN and EGFR*vIII* have a positive response to EGFR inhibitors. In vitro, the coexpression of PTEN and EGFR*vIII* sensitizes GBM cells to Erlotinib [195]. In PTEN-deficient GBM, monotherapy with EGFR small-molecule tyrosine kinase inhibitors is actually inefficient [195,196]. Another study investigated the expression of EGFR, EGFRvIII, Akt, and PTEN in malignant glioma patients treated with Erlotinib. The conclusion was that patients with low EGFR expression and high levels of phosphorylated Akt had a worse outcome after Erlotinib administration [197]. In conclusion, PTEN is an important predictor of RTK inhibitors in gliomas.

PTEN loss also activates mTOR, and it makes sense to use mTOR inhibitors in combination with RTK small-molecule inhibitors to improve the status of GBM patients with PTEN loss [198]. Moreover, PTEN-deficient GBM cells prove to be resistant to therapies when VEGFR-2 is overexpressed.

In recent years, scientists have tried to control PTEN signaling in GBM by using some natural extracts like Icaritin [96], Tangeretin [199], Curcumin [200], and Silibinin [201]. Although these anti-cancer agents proved to have some effect in vitro, they failed in clinical trials [97]. One explanation can be their poor BBB penetrability (Table 1). Also, some of these natural agents (like Icaritin or Tangeretin) are capable of inhibiting GBM progression and metastasis via epithelial–mesenchymal transition (EMT) inhibition. This is a cellular process involved in cancer progression and consists of morphological changes and alterations of the expression of cell adhesion molecules [202]. Both EMT and HIF-1α have very important roles in both the migration and invasion of GBM cells, inducing resistance to treatment [203].

The PI3K/Akt signaling pathway can induce EMT in an hypoxic environment, thereby promoting GBM invasion [204].

### 4.3. Protein Kinase C (PKC) Pathway in Adult Astrocytomas

PKC is a family of serine/threonine kinases which comprises 12 isoenzymes. The classical ones are grouped into three subfamilies, PKCα, PKCβ, and PKCγ, and are activated by diacylglycerol (DAG), calcium, and phosphatidyl serine (PS). Novel PKCs include δ, ε, η, and θ isoforms which only need DAG and PS for activation. GFs are capable of activating PLCβ or PLCγ. Both phospholipases are capable of PIP2 cleavage, which generates DAG but also inositol triphosphate. Inositol triphosphate is capable of releasing calcium from the cells. DAG and calcium activate classical and novel PKCs [205]. Activated PKCs are capable to regulate cell functions like proliferation, differentiation, and apoptosis. In the brain, they regulate the intraneuronal signaling pathways and are also capable of altering the survival and apoptosis of neuronal cells [206].

In cancer, PKC isoforms are capable of regulating cellular processes such as proliferation, migration, and survival via signaling pathways [207]; the Pgp170 pathway, known to be involved in chemoresistance, is also regulated by them.

PKCs are also overexpressed in adult astrocytomas. The classic PKCα stimulates mitosis and therefore proliferation, survival, and also invasion. It is also necessary for the activation of signaling pathways. Being activated by GF like EGF, the isoenzyme is able to link the GF of the mTORC1 pathway, stimulating tumor cell viability. It is also capable of linking bFGF to the ERK1/2 signaling pathway, stimulating tumor cell proliferation [208]. The blockade of PKCα and of the Janus-kinase 2(JAK2) pathway is able to induce apoptosis of GBM cells [209]. PKCβ is involved in adult astrocytoma progression, stimulating angiogenesis because it plays an important role in vessel formation, but other researchers considered that PKCβ has a second quality in these tumors, being able to inhibit GBM tumor growth and increase survival by inducing apoptosis, because it inhibits the hippo pathway [210]. The overexpression of PKCδ in glioma cells downregulates matrix metalloprotease 12, influencing the tumor invasion process. It is also involved in the resistance to treatment by reducing glioma cell sensitivity. PKCδ is also involved in GBM growth and invasion through glycerol-3-phosphate dehydrogenase (GPDH) activation [211]. The involvement of this serine/threonine kinase in the survival and apoptosis of GBM cells, but also in the activation of ERK, determines integrin-dependent glioma cell adhesion and motility [212,213]. In accordance with Aeder et al., in GBM, PKCη targets downstream Akt and mTOR, inducing GBM cell proliferation, differentiation, and apoptosis. Uhr et al. consider that PKCη acts through MEK activation and ERK1 phosphorylation [214]. PKCλ stimulates GBM cell motility and invasion through RhoB, but also GBM cell survival through the PI3K/Akt signaling pathway [215]. PKCζ is associated with gliomagenesis, GBM growth and migration, and tumor invasion [216].

Scientists have concluded that PKC can be a target for adult astrocytoma treatment. For years, several PKC inhibitors have been tested either alone or in combination with other chemotherapeutics in various clinical trials on these patients. The first PKC inhibitor used in a clinical trial for recurrent malignant glioma patients was Tamoxifen, with the results reported in 1995 [217]. Since then, the drug has been tested alone or in combination with other chemotherapeutics like Procarbazine in recurrent high-grade glioma patients and Temozolomide in adult IDH-mutant astrocytomas, in recurrent GB patients, and with radiation therapy in GBM patients, but the results were rather unsatisfactory [218,219]. Enzastaurin, a PKCβ inhibitor, failed to demonstrate its efficacy in a phase III clinical trial for recurrent GBM [220]. Similarly negative results were obtained when it was combined with other inhibitors like Lomustine for GBM patients, temozolomide for glioma patients, bevacizumab for recurrent malignant glioma patients, and radiation therapy for GBM patients [221,222].

### 4.4. JAK/STAT Pathway in Adult Astrocytomas

The JAK/STAT pathway is very important in cell signaling, as it is considered a pole of communication. The JAK family is formed of four members which are non-receptor tyrosine kinases, while the STAT family consists of seven amino acids. The ligands bind the receptor and determine its dimerization, inducing the transphosphorylation and activation of JAK, which determines the transphosphorylation of the bound receptor. This forms a docking site for STAT. At this docking site, JAK phosphorylates STAT and determines its dimerization [223]. This pathway is associated with various diseases including cancer.

In adult astrocytomas, it stimulates tumor growth, invasion, and resistance to treatment, and STAT1 in GBM is considered a poor prognosis factor [224]. Tumorigenesis in GBM is associated with STAT5 which has important roles in tumor proliferation and invasion. Therefore, it is a target for GBM treatment. STAT3 is also overexpressed in gliomas and promotes tumor proliferation, neovascularization, and resistance to apoptosis [225]; the dysregulated STAT3 in GBM activates the connections of the JAK/STAT pathway with other signaling pathways like Notch or NF-Κb signaling. STAT3 is also linked with angiogenesis [226]. It promotes neovascularization through an autocrine feed-forward loop [227]. In addition, STAT3 imbalance in GBM is a result of EGFR amplification, which is encountered in 60% of IDH-wildtype astrocytomas [228]. The aberrant amplification of STAT3 in these tumors is a consequence of the negative feedback regulatory loop of SOCS3, which leads to a reduction in SOCS3 inhibition, further activation of EGFR signaling pathways, and poor prognoses [229]. STAT3 may also promote GBM invasion through matrix-metalloproteinase-2 [230].

Several studies report STAT3 involvement in GBM resistance to therapy [231,232]. One explanation is the redundance and crosstalk between signaling pathways like PI3K/Akt, EGFR, PDGFR, and NF-kB. It has been reported that the administration of Bevacizumab in GBM patients induces STAT3 activation [233]. Despite being an attractive target for GBM therapy, in clinical trials STAT3 inhibitors had unsatisfactory results (Clinical Studies NCT03514069 and NCT02315534). Sorafenib, a Raf, VEGFR2, PDGFR-β, and STAT3 inhibitor, also failed to show any satisfactory benefit for GBM patients in clinical trials [147].

### 4.5. Nuclear Factor Kappa-Light-Chain-Enhancer of Activated B Cells (NF-kB) Pathway in Adult Astrocytomas

NF-κB family members are inducible transcription factors which are involved in cell survival, cytokine production, and so on. The family members are usually dimers and are p65/RelA, RelB, c-Rel, p105/p50 (NF-kB1), and p100/p52 (NF-kB2). The most common NF-kB dimer is the p50/p65 heterodimer [234]. The NF-kB pathway can be activated by various stimuli, including growth factors. They initiate the canonical pathway activation of NF-kB. This activation leads to phosphorylation and activation of the IKK complex. The activated IKK complex, in turn, phosphorylates members of the inhibitor of κB (IκB) family [235]. IκBα, a IkB family member, is targeted for ubiquitin (Ub)-dependent degradation in the proteasome. This process is followed by the release of internuclear gene transcription regulators like Rel-A/p50 or c-Rel/p50 or p50/p50 (only Rel-A/50) [236]. In cancer, NF-κB is involved in tumor initiation through epigenetic changes and the instability of telomerase activity, but also in tumor progression, resistance to apoptosis, and invasion through telomerase reactivation [237]. Moreover, it is a tumor promoter because it is also capable of inducing inflammation.

In adult astrocytomas, the overexpression of GFs causes the dysregulation of the NF-kB signaling pathway. NF-kB signaling enhancement, also stimulated by inflammation, generates the survival of glioma cells but also resistance to treatment. Therefore, it may be associated with tumor aggression and poor outcomes for these patients [238]. For instance, NF-kB activation can be induced by EGFR. The receptor is capable of activating the PKC signaling pathway. PLCγ1 and PKCε monoubiquitylation-dependent IKKβ activation leads to NF-kB activation [239]. Adult astrocytoma cell growth is regulated by NF-kB and controlled by PDFGFR overexpression [240]. In the mesenchymal subtype of GBM, NF-kB has an elevated expression and cooperates with transcription factors such as STAT3 to induce tumor invasion and resistance to treatment. The proneural type of GBM is characterized by PDGFRα amplification and a lower NF-kB activity [241,242]. Moreover, it is already known that NF-Kb pathway activation can be a consequence of both EGFR and c-MET inhibition, and it is followed by the autocrine activation of FGFR which stimulates glial cell survival [104] (Figure 1B).

The overactivation of the NF-kB pathway remains a hallmark of GBM [240], which makes it an attractive target for adult diffuse glioma treatment. Several NF-kB inhibitors such as Sulafasalazine, Bortezomib, Celecoxib, BAY11-7082, and DHMEQ have been tested, either alone or in combination with other therapies, in preclinical studies or phase II clinical studies of patients with adult IDH-mutant or IDH-wildtype astrocytomas. The results were rather modest [243,244,245]. One explanation may be the crosstalk between NF-kB, PI3K, Akt, and mTOR. Compensatory pathways like PI3K/Akt, MAPK, and STAT3 can also be activated as GBM cells can have adaptive resistance [246].

In recent years, a number of natural compounds such as Resveratrol, Quercetin, Sulforaphane, and Curcumin have been studied, mainly in preclinical studies, as NF-kB inhibitors capable of escaping the mechanisms of resistance. Further studies are needed to certify their effect [247,248,249].

### 4.6. Sonic Hedgehog (SHH) Signaling Pathway in Adult Astrocytomas

The SHH signaling pathway is an important regulator of tissue and organ growth during embryonic development [250]. The pathway activation leads to downstream signaling through two mechanisms: canonical and non-canonical. The canonical activation of SHH occurs after the Patched receptor binds the SHH ligand, which is followed by the downstream activation of a signaling cascade. The non-canonical activation of SHH can be realized in two modes. The first one modulates the calcium levels and the actin cytoskeleton, while the second one leads to increased cell proliferation and survival through cyclin B1 [251]. SHH plays an important role in tumorigenesis.

The role of the SHH pathway in adult astrocytoma pathogenesis is widely recognized. SHH signaling is actually amplified by FLT1 (Fms-related tyrosine kinase 1). As we have already mentioned, FLT1 is an RTK which binds its ligand, VEGFA, promoting angiogenesis and also tumor growth. When SHH signaling is suppressed, the consequences of FLT1 overexpression, such as invasion and migration, are also suppressed. As FLT1 promotes glioma angiogenesis, the SHH pathway achieves the same thing through GLI1 [252]. It has also been reported that the SHH pathway is mainly associated with grade 2 and 3 adult astrocytomas. In GBM, the same pathway is less active [253]. GLI1 has interactions with Forkhead Box M1, stimulating glioma growth, survival, and invasiveness, but also with Engrailed 1, which is capable of regulating GLI1 levels, contributing to GBM aggressiveness [254]. Therefore, GLI1 can be an interesting target for adult diffuse glioma patients.

SHH has been reported to interact with other signaling pathways like mTOR. Increased mTOR activity can upregulate GLI1. Therefore, targeting both pathways may be a potential therapeutic option for GBM patients [255]. Another signaling pathway that SHH is correlated with is Wnt, which is also involved in glioma proliferation and invasion [256]. The Notch pathway also interacts with SHH directly through GLI1 and indirectly through Akt, STAT3, and mTOR, contributing to glioma development and progression [257]. The interaction between the PI3K/Akt/mTOR signaling pathway and SHH determines the synergistic stimulation of GBM growth and survival [258].

Drugs targeting the SHH pathway, such as SMO, GLI, SHH, and DNA methyltransferase inhibitors, exhibited some efficiency in preclinical studies but need further research [259].

## 5. Conclusions

The treatment of adults diagnosed with astrocytomas, especially with adult IDH1/IDH2-wildtype infiltrating astrocytic gliomas, is still a challenge for both specialists and patients. The actual standard of care therapy remains the Stupp protocol, first used in 2005, and no other therapeutic option has been able to improve patient status. A better understanding of astrocytoma biology and signaling pathways has helped specialists to understand that the use of a personalized targeted therapy may improve effectiveness compared to classical chemotherapy. Unfortunately, therapies like small-molecule tyrosine kinase inhibitors have failed in clinical trials both for IDH-mutant astrocytomas and for *IDH1*/*IDH2*-wildtype infiltrating astrocytic gliomas. This lack of efficacy has again demonstrated the heterogeneity of adult astrocytomas, the redundance in signal transduction, and their resistance to treatment. We can now conclude that a better characterization of molecular mechanisms of resistance to small-molecule tyrosine kinase treatment in adult astrocytomas, alongside the detection of resistance biomarkers, may be helpful in identifying targeted therapies capable of evading the resistance mechanisms of these tumors.

## Figures and Tables

**Table 1:** Summary of some clinically evaluated kinase inhibitors tested for adult astrocytoma therapy, alongside their BBB penetration and therapeutic responses.

Kinase-Inhibitor Class	Kinase Inhibitor	BBB Penetration	Therapeutic Response
EGFR inhibitors	Erlotinib Gefitinib Cediranib Vandetanib Nintedanib Osimertinib BDTX-1535 ERAS-801	Limited [52] Limited [53] Limited [54] Limited [55] Limited—improved by nanotechnologies [56] Significant [57] Significant [58] Significant [59]	Unsatisfactory [50] Unsatisfactory [60] Unsatisfactory [61] Unsatisfactory [62] Unsatisfactory [63] Unsatisfactory [64] Promising [65] Promising [59]
PDGFR inhibitors	Gleevec Tandutinib	Significant [66] Significant [67]	Unsatisfactory [68,69] Unsatisfactory [70]
VEGFR inhibitors	Cediranib Pazopanib Vandetanib Sorafenib Sunitinib	Limited [54] Limited—can be increased when used in drug combinations [71] Limited—can be increased when used in drug combinations [55] Limited [72] Limited [73]	Unsatisfactory [61] Unsatisfactory [74] Unsatisfactory [75] Unsatisfactory [76] Unsatisfactory [77]
FGFR inhibitors	Infigratinib AZD4547 Debio1347 Erdafitinib LY2874455 Pemigatinib TAS-120	Limited [78]	Unsatisfactory—only Infigratinib has a partial response [79,80]
Tie-receptor inhibitors	Altiranib	Good [81]	Unsatisfactory [82]
MET inhibitors	Cabozantinib Crizotinib	Good [83] Limited [84]	Unsatisfactory [85] In evaluation in clinical trials (NCT04439266181)
MEK inhibitors	Trametinib	Limited [86]	Encouraging but only in combination with Dabrafenib [87]
PI3K inhibitors	Buparsilib PX-866 Paxalisib Bimiralisib	Good [88] Good [89] Good [89] Good [90]	Unsatisfactory [91] Unsatisfactory [92] Unsatisfactory [93] In clinical trial (NCT02850744)
PTEN inhibitors	Icaritin Curcumin	Good [94] Limited—can be improved by nanocarriers [95]	Modest [96] Unsatisfactory [97]

## Data Availability

No new data were created or analyzed in this study. Data sharing is not applicable to this article.
